# Power of “Accommodation” in the Human Eye; Its Physiology and Pathology

**Published:** 1858-12

**Authors:** E. L. Holmes


ARTICLE II.

### POWER OF "ACCOMMODATION" IN THE HUMAN EYE; ITS PHYSIOLOGY AND PATHOLOGY.

BY E. L. HOLMES, M. D.

Scarcely any question in the physiology of vision has offered greater difficulties in its solution, than that relating to the adjustment of the eye to different distances. Anatomists have dissected the eye and studied its minutest textures, and physiologists have invented theories by which to explain this wonderful and beautiful endowment. But with all these dissections and all their theories, they have till quite recently *established* scarcely one fact.

Reasoning upon the general principles of optics, and comparing the eye with optical instruments, we may undoubtedly state what alterations *may* take place in the eye, to account for variations in its focal distance. There is certainly nothing improbable in the theory, that this may be produced—1st, by increasing or diminishing the antero-posterior diameter of the crystaline lens; 2d, by increasing or diminishing the distance between the retina and the lens; or, 3d, by the united effects of both these changes. But what actually does take place is a question which has long interested the physiologist, and which will probably long be discussed before it is wholly decided. This difficulty arises, in part, from the delicate and complex structure of the eye itself; the various portions of which are so easily changed in relative position by examination, that scarcely any two anatomists agrée in their delineations of the eye. We can convince ourselves of this by reading the different descriptions and comparing the form and position of different portions of the organ, as given in the numerous anatomical works of the present age.

The eye has been carefully divided and studied when frozen, when coagulated by certain chemical substances, and, when prepared, by being first divided in a dried state, and then allowed to resume as near as possible its normal shape by the absorption of water. By these various modes of investigation, much progress has been made in the minute anatomy of the eye. Still, in studying the function of "accommodation" to different distances, most observers have been too prone to theorize upon the use of each particular portion of the eye, without first having learned the changes which actually take place within it, some of which we shall show can be made visible to the observer during the act of adjustment.

The mode of investigation adopted by Cramer of Berlin, Donders of Utrecht, and Helmholz of Konigsberg, is more satisfactory than others, since it clearly *demonstrates* some of the changes which were only supposed by former physiologists to occur in the eye. The experiments of these physiologists were based upon the fact that the images of an object produced by convex or concave mirrors, stationed *at a given distance* from the object, varied in size according to the degree of convexity or concavity of the mirrors.

Helmholz, by a long series of experiments with very delicate and expensive apparatus, was not only able to measure the length of the images of a particular object formed by the cornea, the anterior convex and posterior concave surfaces of the lens, but also to calculate the differences in degree of convexity which appeared in the lens in different states of the age. He was also able to measure changes in the relative distances between the cornea, anterior and posterior surface of the lens.

This instrument, which has been called the ophthalmometer, has been used by the most celebrated experimental physiologists in obtaining the measurements of the eye, which are now considered as most accurate. By means of these measurements, and the formulæ used by Brewster, Krause and other opticians in calculating the refracting power and curves of the different media of the eye, Helmholz and others have been enabled, as I

just stated, to establish certain facts regarding the crystaline lens, which have a very important bearing on the question before us.

The movements of the iris, at the moment the focus of the eye is adjusted for a particular object, had long before been noticed.

Whenever the eye is fixed upon a near object, the following variations are observed:

1*st.* The pupil contracts.

2*d.* The pupilary edge of iris is thrown forward.

3*d.* The outer portion of the iris recedes.

4*th.* The anterior convex surface of the lens is rendered more convex, and at the same time is thrown forward.

5*th.* The convexity of the posterior surface of the lens is but little increased, and is scarcely changed in position.

We thus see that the refractive power of the lens is increased.

The contour of the cornea is not affected by the ordinary acts of accommodation, since it is shown by the apparatus of Helmholz, that the image of an object, formed upon its surface, unlike those formed upon the anterior and posterior surfaces of the lens, suffers no modification whatever.

In turning the eye from a near to a remote object, the reverse phenomena are observed; that is, the pupil dilates—the pupillary margin of the iris recedes, while its external portion slightly advances, and the antero-posterior diameter of the lens, as also its convexity, is diminished.

These facts are now received by many of the best authorities as fully established, and they are certainly facts of great interest to the student of science. Although many physiologists are united in opinion as regards the facts, they are by no means disposed to agree concerning the manner in which the changes, above mentioned, are produced, and consequently several theories have been proposed to account for them. Perhaps that of H. Muller, as stated in the *Archiv. fur Ophthalmogie*, vol. iii., is the theory most consistent with the anatomy of the anterior portion of the eye. Prof. Arlt, however, after most careful dissection of more than 300 eyes, prepared in various ways, delineates the anterior portions of the eye somewhat differently from Muller, as also from Helmholz and Brucke, and differs from these physiologists in explaining the manner in which adjustment is produced.

It is well known, that Brucke and Bowman proved that the ciliary muscle, which lies outside the vitreous humors, between the anterior limits of the retina and the union of the selerotica with the cornea, is composed of two series of muscular fibres—the radiating, which pass from the anterior termination of the selerotica towards the ora serata of the retina, and the annular fibres, which pass in a circle parallel to the circumference of the cornea, forming a kind of sphincter, similar to the corresponding fibres of the iris.

In addition to these muscular fibres, there are others, composing the zonule of Zinn, which appears to be a continuation of the retina, modified however in structure. The function of this organ has long been a subject of discussion among physiologists. It is now regarded by many of them as the antagonist of the ciliary muscle.

Muller explains the action of these fibres in the adjustment to near objects as follows:

1*st*. The annular fibres, in contracting, exert pressure upon the periphery of the lens, which increases the length of its antero-posterior diameter—that is, makes it thicker and more convex.

2*d*. The radial or longitudinal fibres, in contracting, tend to draw the choroid coat, and, consequently, the vitreous humor, forward, thus preventing the posterior portion of the lens from receding at the moment the diameter of the lens is increased as just described.

3*d*. The pressure of the external portion of the iris upon the corresponding portion of the capsule of the lens, tends to increase its convexity anteriorly.

4*th*. The contraction of the ciliary muscle produces a relaxation of the zonule of Zinn, which relaxation also favors an increase in the diameter of the lens.

5*th*. The contraction of the zonule, supposed to occur while the ciliary muscle is in a state of relaxation, draws literally upon the capsule, thus rendering the lens less thin, and, consequently, less powerful.

The changes, which have been proved to exist in the lens, and the situation of the muscular fibres, are such, that they *seem* to bear the relation to each other of cause and effect. This theory derives support from facts in comparative anatomy. It is stated by several authorities that these muscles are developed to a wonderful degree in those birds which are remarkable for their great power of accommodation. The lens in such birds is also of less consistency than in many other animals, which renders modifications in its form more readily effected.

The contraction of the pupil, during the act of accommodating the focus of the eye to near objects, is probably of less importance than the other phenomena we have noticed, since we know that the changes, produced by ordinary variations in the amount of light entering the eye, do not modify the power of adjustment. A still stronger proof, that the adjustment of the focus may take place independent of the action of the iris, is found in cases in which paralysis of the iris and the loss of adjusting power are observed in the same eye, as reported by Graefe. After treatment, in one case the function of accommodation was restored, while the paralysis of the iris remained; in another case, the paralysis was cured without any effect upon the loss of accommodation, showing that the two functions to a certain extent, at least, are independent of each other. Even without the good result of treatment, such cases would afford no argument against our supposition, since the same cause, which produced the paralysis of the iris, operating upon nerves in so close proximity, might also produce the paralysis of the ciliary muscle and the zonule.

DesMarais reports a case of mydriasis following a blow near the eye, in which the power of adaptation remained perfectly normal.

It has been supposed that the recti and obliqui muscles of the eye, had also some influence in the adjustment of focal distance. An argument for this opinion is thought to be found upon the fact that very often, after the operation for strabismus, vision is improved. But that accommodation may be entirely independent of these muscles, is proved by a case reported by Graefe, in which all twelve muscles of the eyes were completely paralyzed, and still the power of adjustment was perfectly intact. It is true, *sight* is improved by the operation for strabismus, but this is probably independent of any modification in the function we are now considering. We must remember that an eye affected with strabismus often becomes amblyopic in consequence of disease, the patient finding, by experience, that he is unable to see distinctly with the eccentric portion of the retina. The organs of adjustment become also weakened from disease. Now, an operation, by restoring the eye to its normal position, also restores the central point of the retina in harmony with the other eye, whereby this portion of the nerve and also the muscles of adjustment, by performing their normal function, regain their lost power. That a muscle of the eye in disease, by undue pressure upon the globe, *may* affect the focal distance of the eye, is undoubtedly true, but whether in health this is actually the case, is scarcely probable. There is more reason to believe that the orbicularis *may* assist the eye in changing its focal distance, since the near-sighted involuntarily contract this muscle as much as possible in viewing distant objects. Because a muscle *may* possibly perform a certain act in certain abnormal conditions of muscles near it, it does not follow that it does it habitually in health any more than that the muscles of the *arm* assist the eye in "accommodation," because a very near-sighted person, by pressing gently upon the eye with his finger, *may* render the organ somewhat more far-sighted.

On the same principle, the argument, founded upon the fact that a patient occasionally preserves, to a considerable extent, the power of adaptation, even after the double operation for cataract by extraction, is really of little weight; since such a case is quite rare, and only proves that *some* persons can employ the other muscles of the eye to assist it in adjusting its focus, just as we *occasionally* find an individual who has a remarkable power over the muscles of the ear (auricle) and scalp.

In the present state of anatomy and physiology of the eye, therefore, we may regard the following as the most essential points in the theory of accommodation:

1*st*. The focus is adapted to near objects by an increase in the thickness and convexity of the lens, and by a slight motion of the lens from the retina; to distant objects, by a diminution in the thickness and convexity of the lens, and a slight motion of the lens *towards* the retina.

2*d*. The former of these changes are produced by the ciliary muscle, and the latter by the zonule of Zinn.

The nerves which supply the ciliary muscle and the zonule of Zinn have not as yet been satisfactorily demonstrated. They, however, like the muscular fibres themselves, are probably entirely independent of the iris and its nerves.

Like every organized structure, the portions of the eye which we have thus far considered are liable to disease. The pathology of these diseases, as well as the physiology of the function itself, has been enveloped in obscurity. But since it has been well understood that "accommodation" depends principally upon a particular series of muscular fibres, an explanation of numerous morbid conditions is readily found.

The most frequent example of abnormal condition of the muscles of "adaptation" is offered in very many cases of myopia and presbiopia, or far and near sightedness, although, in a vast number of instances, we can find no cause of these affections, except in some idiosyncrasy in the individual, in modifications of the consistency of the lens, or in changes produced by advancing age; yet there are thousands of people who have rendered themselves far or near sighted by an injudicious use of their eyes.

The ciliary fibres, as also those of the zonule, are, to a certain extent, developed in proportion to the exercise to which they are subjected. Hence, we see that, as a general rule, those persons who employ their sight equally upon near and distant objects, usually suffer little from difficulties of adjustment. On the other hand, the young who are confined within the walls of the schoolroom, who spend much time over books, and who rarely look beyond the narrow lanes of the city, by constant exercise of the ciliary muscle have so little demand upon the zonule that it at last loses much of its power. The consequence of this is, that the lens is so constantly confined in a position adapted to near objects, that efforts to look at a distance are followed with pain. Regarding the ciliary muscle as an analogue of the flexor muscles, and the zonule of the extensors, we can compare the condition of the eye just described to that of an arm which has been retained in a state of flexion, efforts to extend which are accompanied with discomfort.

The sailor, who spends so many hours in the open air, looking at distant objects, has little exercise for the ciliary muscle, while the zonule is ever in a state of action. This condition may be compared to that of an arm kept long extended. Efforts to flex it are painful.

The eyes often become myopic when affected with incipient amaurosis. To obtain as distinct an image of an object as possible, a greater quantity of light than usual must enter the pupil; to effect this, the object must be brought near the eye, and the focus of the lens is adjusted accordingly. In time, the habit of placing the eye so near an object is confirmed, and the ciliary muscle becomes abnormally developed. Great care is necessary in the selection of glasses for patients who have partially lost the power of accommodation, otherwise they aggravate the evil for which they are adopted. Under ordinary circumstances, a convex glass, more powerful than is required, renders the eye more *presbiopic*, and a too powerful concave glass renders it more *myopic*.

Besides the common affections of far and near-sightedness, there are several others which, although not so frequent, are by no means rare.

I have lately had occasion to examine two individuals whose eyes, to all appearances, were perfect. They had the power of adjusting, with great facility, the foci from a near and minute object to a distant one, so that they could read the finest print and distinguish small objects at a very great distance off. And yet these individuals were wholly unable to follow their vocations, which required their eyes constantly fixed upon minute objects. After reading a few moments the sight grew dim, accompanied with pain over the brow and in the globe itself. The affection in each of these cases is probably one implicating the ciliary muscle. For some reason this muscle is unable to support a continued state of traction and soon tires. This state of the eye has been called asthenopia, and is a very common disease in the weak and debilitated, who are obliged to employ their eyes long at a time upon minute objects with insufficient light. Such cases are usually complicated with slight congestion of the retina and choroid. Cases like the two which I have just cited, without complication, are less frequent.

Slight blows on or near the eye, or injuries of the fifth pair of nerves, sometimes produce less of the power of accommodation. A patient with such an injury may be able to see an object placed at a given distance, but has no power to distinguish objects with accuracy at a greater or less distance. Graefe reports several cases in which loss of accommodation was caused by very slight injuries.

A singular condition of the eye is occasionally found in which a person who, within certain limits, is by no means near-sighted, is yet unable to distinguish distant objects. For instance, a patient may be able to read the finest print at the distance of a couple of feet, and ordinary type at the distance of four or five feet, and not be able to distinguish the sashes of a window at the distance of fifty feet. A near-sighted person who could not read the same print except by bringing it much nearer the eye, could much more easily distinguish the different portions of the window. A wholly satisfactory explanation of this peculiarity has not yet been offered, although the muscles of adaptation are probably implicated.

I am well aware that I have omitted several questions of interest connected with this subject, but they are of minor importance, and can scarcely strengthen the arguments for or against the principles I have discussed.

